# Synthesis of Zearalenone Immunogen and Comparative Analysis of Antibody Characteristics

**DOI:** 10.1155/2021/7109383

**Published:** 2021-07-26

**Authors:** Yanan Wang, Xiaofei Wang, Haitang Zhang, Jinqing Jiang, Hanna Fotina

**Affiliations:** ^1^College of Animal Science and Veterinary Medicine, Henan Institute of Science and Technology, Xinxiang 453003, China; ^2^Faculty of Veterinary Medicine, Sumy National Agrarian University, Sumy 40021, Ukraine; ^3^Xinke College, Henan Institute of Science and Technology, Xinxiang 453003, China

## Abstract

**Background:**

This study aimed to explore the zearalenone (ZEN) immunogen synthesis method, immunogenicity, and antibody characteristics and to lay a foundation for the establishment of immunoassay methods for ZEN single residue and ZEN and its analogs total residue.

**Methods:**

Based on the molecular structure and active sites of ZEN, oxime active ester (OAE), condensation mixed anhydride (CMA), formaldehyde (FA), and 1,4-butanediol diglycidyl ether method (BDE) were designed and used for immunogen (ZEN-BSA) synthesis. The immunogens were identified by infrared (IR) and ultraviolet (UV) spectra and gel electrophoresis (SDS-PAGE) and were then used to immunize Balb/c mice to prepare ZEN polyclonal antibody (ZEN pAb). The titers and sensitivity of the ZEN pAb were determined by indirect noncompetitive ELISA (inELISA) and indirect competitive ELISA (icELISA), respectively, and its specificity was assessed by the cross-reaction test (CR).

**Results:**

ZEN-BSA was successfully synthesized, and the molecular binding ratios of ZEN to BSA were 17.2 : 1 (OAE), 14.6 : 1 (CMA), 9.7 : 1 (FA), and 8.3 : 1 (BDE), respectively. The highest inELISA titers of ZEN pAb of each group were 1 : (6.4 × 10^3^) (OAE), 1 : (3.2 × 10^3^) (CMA), 1 : (1.6 × 10^3^) (FA), and 1 : (1.6 × 10^3^) (BDE), respectively. The 50% inhibition concentrations (IC50) for ZEN by icELISA of each group were 11.67 *μ*g/L (OAE), 16.29 *μ*g/L (CMA), 20.92 *μ*g/L (FA) and 24.36 *μ*g/L (BDE), respectively. ZEN pAb from the mice immunized with ZEN-BSA (OAE) and ZEN-BSA (CMA) had class broad specificity to ZEN and its analogs. The CRs of ZEN pAb with *α*-ZAL, *β*-ZAL, *α*-ZOL, *β*-ZOL, and ZON were 36.53%, 16.98%, 64.33%, 20.16%, and 10.66%, respectively. ZEN pAb from the mice immunized with ZEN-BSA (FA) and ZEN-BSA (BDE) had high specificity for ZEN. The CRs of ZEN pAb with its analogs were all less than 1.0%.

**Conclusion:**

This study demonstrated that the preparation of the class broad-specificity antibodies of ZEN and its analogs can be achieved by immunizing animals with the immunogen ZEN-BSA prepared by the OAE method, while the preparation of highly specific antibodies can be achieved by immunizing animals with the immunogen ZEN-BSA prepared by the FA method. These findings lay the material and technical foundation for immunoassay of ZEN single residue and ZEN and its analogs total residue.

## 1. Introduction

**Figure 1:**
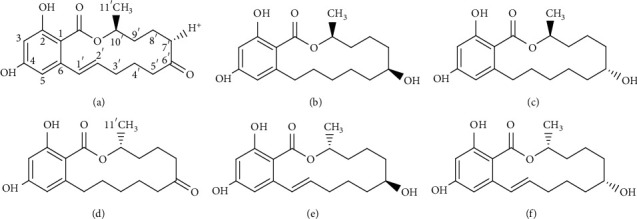
The chemical structure of zearalenone and its analogs. (a) Zearalenone (ZEN). (b) Alpha-zearalanol (*α*-ZAL). (c) Beta-zearalanol (*β*-ZAL). (d) Zearalanone (ZON). (e) Alpha-zearalenol (*α*-ZOL). (f) Beta-zearalenol (*β*-ZOL).

Zearalenone (ZEN) is a toxic secondary metabolite produced by members of the genus *Fusarium*, and ZEN mainly contaminates grains including corn, wheat, barley, rice, and oats or foods containing these grains, and its chemical name is (E)-(S)-2,4-dihydroxy-7-methyl-7,8,9,10,13,14-hexahydro-12h-6-oxa-benzocyclotetradecene-5,11-dine, also called F-2 toxin. The ZEN analogs produced under natural conditions mainly include *α*-zearalanol (*α*-ZAL), *β*-zearalanol (*β*-ZAL), *α*-zearalenol (*α*-ZOL), *β*-zearalenol (*β*-ZOL), and zearalanone (ZON) (Figure 1) [1, 2]. Owing to the reproductive toxicity, genotoxicity, immunotoxicity, endocrine toxicity, and carcinogenic toxicity of ZEN to the body [3, 4], most countries and regions globally have implemented the maximum residue limits (MRLs) of ZEN in food and feed. The EU stipulates that the MRL of ZEN in cereals and grain products is 2 mg/kg; the MRL of ZEN in corn by-products is 3 mg/kg; the MRL of ZEN in compound feeds for piglets and young sows are 0.1 mg/kg [5]. For instance, Italy stipulates that the MRL of ZEN in grains and cereal products is 0.1 mg/kg [6]; Australia stipulates that the MRL of ZEN in grains is 0.05 mg/kg [7]. The current ZEN MRL standard of food and agricultural products in China is “GB 2761-2017 Food Mycotoxin Limit,” which strictly stipulates that the MRL of ZEN in wheat, wheat flour, corn, and corn flour is 0.06 mg/kg [8]. However, with people's increasing attention to food safety issues, researchers have extensively explored the occurrence and toxicity of ZEN and its analogs. Reports show that ZEN, *α*-ZOL, and *β*-ZOL often exist simultaneously in cereals infected by *Fusarium spp.* Notably, ZEN can be metabolized into *α*-ZOL, *β*-ZOL, and ZON. *α*-ZAL is the reducing product of ZEN and is metabolized into *β*-ZAL and ZON. Thus, ZEN and its analogs are similar, with toxic effects on the human body. However, *α*-ZOL has the strongest toxicity, which is 10–20 times higher than ZEN [9, 10]. Therefore, single ZEN detection cannot meet the needs of food and feed industry, and this has prompted immense research on detecting the total amount of ZEN and its analogs.

Currently, there are two main methods for detecting ZEN residues, including physicochemical analysis and immunoassay. The main physicochemical analysis methods used in all countries are thin-layer chromatography (TLC) [11], high-performance liquid chromatography (HPLC) [12], gas chromatography-mass spectrometry (GC-MS) [13], and liquid chromatography/tandem mass spectrometry (LC-MS/MS) [14], among others. However, these techniques are expensive and lengthy and require complicated sample pretreatment procedures, expensive instruments, and skilled technicians. Therefore, they are not good enough to meet the needs of actual detection [15]. The immunoassay methods based on antigen-antibody reaction have the common characteristics of strong selectivity and high sensitivity and have become a hot topic in ZEN detection research, such as flexible pressure sensor (FPS) [16], self-powered temperature sensor (SPTS) [17], nano-hybrid-mediated photoelectrochemical immunoassay (NMPECIA) [18], and quantum dot-based photoelectrochemical immunoassay (QDPECIA) [19]. In addition, the established immunoassay methods, such as enzyme-linked immunosorbent assay (ELISA) [20], gold immunochromatographic assay (GICA) [21], and the time-resolved fluoroimmunoassay (TRFIA) [22], also have the advantages of fast, low cost, and on-site detection [15], fluorescence polarization immunoassay (FPIA) [23], immunosensor (IS) [24], and antibody microarray (AMA) [25] have the advantage of the high-throughput detection. These immunoassay techniques play an important role in the rapid detection of ZEN. However, there are associated drawbacks such as poor specificity in single ZEN immunoassay and poor broad-spectrum in total detection of ZEN and its analogs, which cannot meet the actual needs of detection. Such challenges may be attributed to low quality of the prepared antibody. Of note, high-quality antibodies are the core reagents for establishing immunoassay methods, and the production of highly specific antibodies and class broad-specificity antibodies depends on the design of hapten molecules and immunogen synthesis [26].

At present, there have been many reports on the research of ZEN antigen synthesis, antibody preparation, and immunoassay method establishment, but there are no reports about the research on the comparison and analysis of ZEN different antigen synthesis and antibody characteristics. In view of this, through the molecular design of ZEN hapten, immunogen synthesis and identification, preparation and characteristic comparison, and analysis of polyclonal antibody (pAb), we selected the best immunogen synthesis method, which laid the foundation for the specific single residual immunoassay of ZEN and the total amount residual immunoassay of ZEN and its analogs (Figure 1).

## 2. Materials and Methods

### 2.1. Chemicals and Reagents

ZEN standard (solvent-free), *α*-zearalanol (*α*-ZAL), *β*-zearalanol (*β*-ZAL), *α*-zearalenol (*α*-ZOL), *β*-zearalenol (*β*-ZOL), and zearalanone (ZON) standard solutions in methanol, carboxymethoxylamine hemihydrochloride (CMO), N-hydroxysuccinimide (NHS), N-(3-dimethylaminopropyl)-N′-ethyl-carbodiimide (EDC), hydroxylamine hydrochloride, succinic anhydride, 1,4-butanediol diglycidyl ether (BDE), phenacetin, 3,3,5,5-tetra-methylbenzidine (TMB), urea peroxide, and Tween-20 were purchased from Sigma-Aldrich (St. Louis, MO, USA). Bovine serum albumin (BSA), ovalbumin (OVA), Freund's complete adjuvant (FCA), Freund's incomplete adjuvant (FIA), and goat anti-mouse IgG conjugated with horseradish peroxidase (GaMIgG-HRP) were obtained from Pierce (Rockford, IL, USA). Dioxane, isobutyl chloroformate (IBCF), dimethylformamide (DMF), and formaldehyde (FA) were from Beijing Chemical Works (Beijing, China). Meanwhile, ethylenediamine (EDA) and tri-n-butylamine (TNBA) were from J&K Chemicals (Shanghai, China). All other solvents, reagents, and chemicals were standard commercial products of analytical grade or better.

### 2.2. Standard Solutions and Buffers

The standard solutions used were as follows: (1) stock solutions of ZEN, *α*-ZAL, *β*-ZAL, *α*-ZOL, *β*-ZOL, and ZON at a concentration of 1000 ng/mL were prepared in methanol and diluted to a standard solution with 0.01 mol/L PBS; (2) cationized carrier protein solution, 200 mg (0.003 mmol) BSA (or 135 mg, 0.003 mmol, OVA), was dissolved in 10 mL PBS, and 7.2 mg (0.12 mmol) EDA and 11.5 mg (0.06 mmol) EDC was added sequentially. The solution was magnetically stirred at room temperature for 2 h. The reaction solution was dialyzed with PBS at 4°C for three days, and the dialysate was changed once a day to obtain cationized BSA (cBSA) or cOVA, stored at 4°C for subsequent use.

The buffers used included the following: (1) phosphate buffer saline (0.01M PBS, pH 7.4), consisting of NaCl (137 mmol), Na_2_HPO_4_·12H_2_O (10 mmol), KCl (2.68 mmol), and KH_2_PO_4_ (1.47 mmol); (2) for the coating buffer, carbonate buffer (0.05 M CBS, pH 9.6), consisting of Na_2_CO_3_ (15 mmol) and NaHCO_3_ (35 mmol); (3) washing buffer consisting of PBS, containing 0.05% Tween-20 (PBST); (4) blocking buffer containing swine serum (5%, v/v) in PBST; (5) substrate buffer made from a mixture of part A (500 mL) and part B (500 mL) solutions. Part A contained (per 1 L water) 3.15 g citric acid, 6.966 g anhydrous sodium acetate, 0.08 g phenacetin, and 0.05 g urea peroxide adjusted to pH 5.0 with HCl. In contrast, Part B contained 1.27 g TMB dissolved in 500 mL methanol and 500 mL glycerol. (6) We used 2 M H_2_SO_4_ as the stopping solution.

### 2.3. Equipment and Instruments

A spectrophotometric microtiter reader (Multiskan MK3, Thermo Co., Shanghai, China) was used to measure the absorbance. The infrared (IR) spectra were obtained using a Bruker Tensor 27 spectrometer (Bruker, Germany), while the ultraviolet (UV) visible spectra were obtained with a DU-800 UV visible spectrophotometer (Beckman-Coulter, Fullerton, CA, USA). JY300C electrophoresis apparatus and gel imaging system were purchased from Beijing Junyi Dongfang Electrophoresis Equipment Co., Ltd. (Beijing, China). In addition, a 303A-1 electric heating constant temperature incubator was purchased from Beijing Zhongxing Weiye Instrument Co., Ltd. (Beijing, China). Exceed DZG-303A ultrapure water polishing system was purchased from Chengdu Kangning Special Experiment Pure Water Equipment Factory (Chengdu, China). An LDZX-30KB vertical pressure steam sterilizer was purchased from Shanghai Shenan Medical Instrument Factory (Shanghai, China). Transparent 96-well polystyrene microtiter plates were from Boyang Experimental Equipment Factory (Jiangsu, China).

### 2.4. Experimental Animals

Six-week-old female Balb/c mice and feed were provided by the Experimental Animal Center of Medical College of Zhengzhou University (Zhengzhou, China). They were given tap water and a diet *ad libitum*. The room housing the animals was maintained at a temperature of 24 ± 2°C, relative humidity of 50% ± 20%, and a 12 h light/dark cycle.

### 2.5. Synthesis of Immunogen

According to the molecular structure and active site of ZEN, the immunogen and coating antigen were synthesized via oxime active ester (OAE) [27], condensation mixed anhydride (CMA) [28], formaldehyde (FA) [29], and 1,4-butanediol diglycidyl ether (BDE) techniques [30].

**Figure 2:**
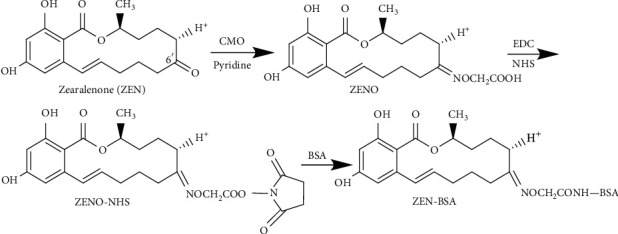
Synthesis route of the ZEN-BSA by the OAE method.

The basic principle of the OAE method was as follows. The C6′ position carbonyl group of ZEN molecule was selected as the active site, and ZEN oxime (ZENO) was synthesized by oximation reaction with CMO. The carboxyl group of ZENO and amino group of BSA were coupled with a monoamide bond under the action of EDC to synthesize artificial immunogen ZEN-BSA. Briefly, according to the initial molar ratio of ZEN and BSA (50 : 1), 5 mg (0.0157 mmol) ZEN was dissolved in 2 mL pyridine, followed by the addition of 10 mg (0.09117 mmol) CMO. After sealing, the reaction proceeded while stirring using a magnetic bar at room temperature for 24 h to obtain the yellow solution product. The product was dried using nitrogen, and then, we added 3.0 mL deionized water, adjusted pH to 8.0 using 0.1 mol/L NaOH, and extracted with an equal volume of ethyl acetate thrice. The aqueous phase was discarded. The organic phase was collected and dried using nitrogen to obtain hapten ZENO, which was a light yellow oil. ZENO was dissolved in 2 mL dioxane, after which 2.5 mg NHS and 5 mg EDC were added, and stirred at 4°C for 4 h to prepare the hapten activation solution. To the hapten activation solution, 1 mL cBSA activation solution was added at a concentration of 20 mg/mL dropwise. The reaction solution was stirred at 4°C for 4 h and then dialyzed with PBS at 4°C for three days. The dialysate was changed once a day and stored at 4°C for subsequent applications. The synthetic route of ZEN-BSA (OAE) is shown in Figure 2. The coating antigen ZEN-OVA (OAE) was prepared via the same method.

**Figure 3:**
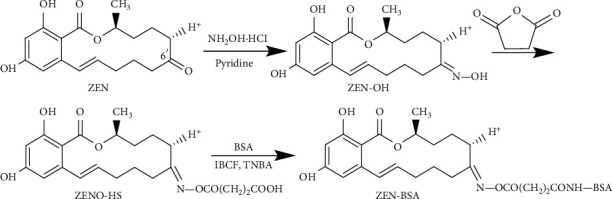
Synthesis route of the ZEN-BSA by the CMA method.

The basic principle of the CMA method was as follows. The C6′ position carbonyl group of ZEN molecule was selected as the active site, and the hydroxyl group was introduced via condensation with hydroxylamine hydrochloride. The hydroxyl group reacted with succinic anhydride to introduce the active group carboxyl group. The carboxyl groups react with isobutyl chloroformate in the presence of tri-*n*-butylamine to generate active intermediate mixed anhydride, which then reacts with primary amino groups on protein carriers to release an immunogen by monoamide coupling. Briefly, according to the initial molar ratio of ZEN and BSA (50 : 1), 5 mg (0.0157 mmol) ZEN was dissolved in 2 mL pyridine, and 5.5 mg (0.0785 mmol) of hydroxylamine hydrochloride was added. The reaction was stirred at room temperature for 24 h, after which pyridine and unreacted hydroxylamine hydrochloride were removed via rotary evaporation to obtain the primary product. The initial product was dissolved in 0.5 mL pyridine, and 7.8 mg (0.785 mmol) succinic anhydride was added, followed by 1 mL tetrahydrofuran. The reaction was stirred at room temperature for 24 h. The solvent was removed via rotary evaporation to obtain the hapten product, which was dissolved in 2 mL of dioxane, and then, 15 *μ*L of tri-n-butylamine and 45 *μ*L of isobutyl chloroformate were added. The reaction was stirred for 0.5 h at 0°C in an ice bath. Eventually, the hapten activation solution was formed, which was added dropwise to 1 mL of cBSA activation solution at a concentration of 20 mg/mL. The reaction was stirred at 4°C for 4 h. We dialyzed the reaction with PBS at 4°C for three days. The dialysate was changed once a day. The resulting ZEN-BSA conjugate was stored at 4°C for subsequent use. The synthetic route of ZEN-BSA (CMA) is shown in Figure 3. Notably, the coating antigen ZEN-OVA (CMA) was prepared via the same method.

**Figure 4:**
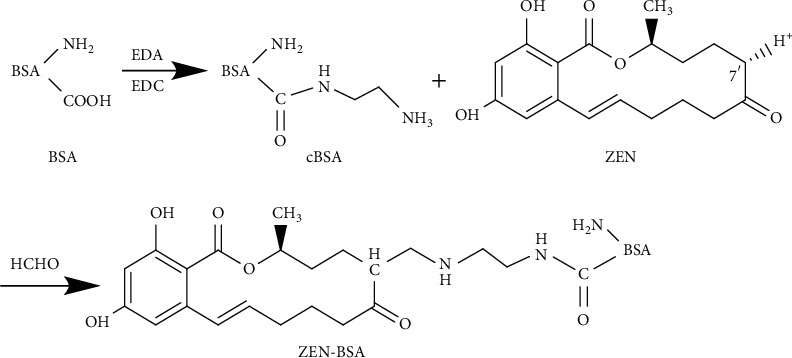
Synthesis route of the ZEN-BSA by the FA method.

The basic principle of the FA method was as follows: the artificial immunogen was synthesized by the Mannich condensation reaction between the hapten containing active hydrogen and the amino group of protein triggered by formaldehyde. We used the active hydrogen at the C7′ position of ZEN as the active site. Briefly, the experiment was designed according to an initial molar ratio of 50 : 1 (ZEN to BSA). Exactly 5 mg (0.0157 mmol) ZEN was dissolved in 1 mL of dimethylformamide (DMF) and then added dropwise to 1 mL at a concentration of 20 mg/mL cBSA activation solution. 60 *μ*L 37% formaldehyde aqueous solution (containing formaldehyde 0.8 mmol) was added, and the reaction was magnetically stirred at room temperature for 24 h. The reaction solution was dialyzed with PBS at 4°C for three days, and the PBS was changed once a day. The dialysate was collected and stored at 4°C for subsequent use. The synthetic route of ZEN-BSA (FA) is shown in Figure 4. The coating antigen ZEN-OVA (FA) was prepared via the same method.

**Figure 5:**
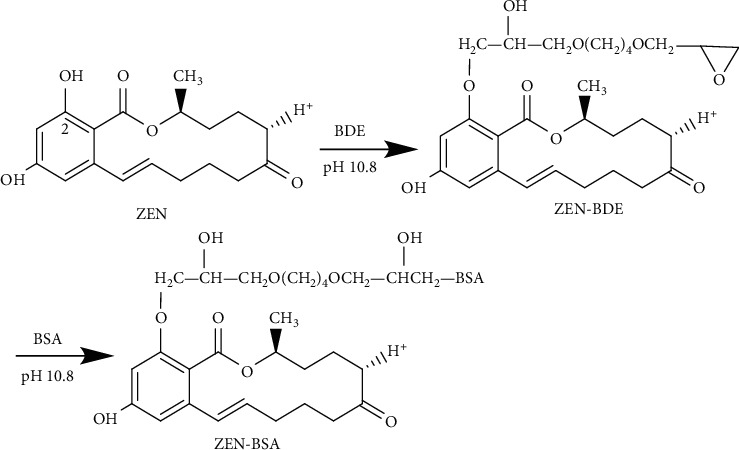
Synthesis route of the ZEN-BSA by the BDE method.

The basic principle of the BDE method was as follows: using the active site of the C2 position hydroxyl group on the ZEN molecule, 1,4-butanediol diglycidyl ether contains an oxirane group at both ends. One end of the oxirane group reacts with the hydroxyl group of the hapten to form an ether bond. At the other end, the oxirane group reacts with the amino group of the carrier protein to form a secondary amine, which is coupled to synthesize an artificial immunogen through the BDE long-chain bond. Briefly, the experiment was designed according to the initial molar ratio of 50 : 1 (ZEN to BSA); 5 mg (0.0157 mmol) ZEN was dissolved in 0.5 mL dimethylformamide (DMF); 31 *μ*L (0.157 mmol) 1,4-butanediol diglycidyl ether (BDE) was dissolved in 0.5 mL of double-distilled water and added dropwise to the previously mentioned solution. Then, the pH was adjusted to 10.8 with 1 mol/L NaOH, and the solution was magnetically stirred at room temperature for 4 h. This formed the hapten activation solution. The hapten activation solution was added dropwise to 1 mL of cBSA activation solution with a 20 mg/mL concentration, the pH was adjusted to 10.8 with 1 mol/L NaOH, and the reaction was stirred at 4°C for 4 h. The reaction solution was dialyzed with PBS at 4°C for three days. The dialysate was changed once a day. After collection, the dialysate was stored at −20°C for subsequent use. The synthetic route is shown in Figure 5. The coating antigen ZEN-OVA (BDE) was prepared via the same method.

### 2.6. Identifying the Artificial Immunogen

#### 2.6.1. IR Identification

Exactly 2 mg of freeze-dried immunogen was mixed with an appropriate amount of potassium bromide, grounded evenly, press tableted, and tested on a Bruker Tensor 27 spectrometer [31].

#### 2.6.2. UV Identification

PBS was used as blank control to calibrate the baseline. We prepared a 1 mg/mL BSA solution and diluted ZEN-BSA to a 1 mg/mL protein concentration. The concentration of the ZEN standard was made to 20 *μ*g/mL. We did a scan within the wavelength range of 200 to 800 nm, analyzed the scanning spectrum, and calculated the molecular binding ratio of ZEN to BSA [32].

#### 2.6.3. SDS-PAGE Identification

Here, 5% concentration gel and 12% separation gel were selected for electrophoresis analysis. The test voltage for the concentrated gel was 100 V, while that for the separation gel was 60 V. Moreover, 10 *μ*L/hole sample volume and 10 *μ*g/hole protein content were used. After the Coomassie brilliant blue staining, the molecular binding ratio of ZEN and BSA was calculated by ultraviolet gel imaging system analysis software [33].

### 2.7. Preparation of ZEN pAb

Four immunogens of ZEN-BSA (OAE), ZEN-BSA (CMA), ZEN-BSA (FA), and ZEN-BSA (BDE) were used to immunize Balb/c in four groups and five mice in each group. The immunization method was subcutaneously injected into the neck at multiple sites. The immunization dose was 50 *μ*g/head, calculated according to the amount of BSA in ZEN-BSA. Mice were immunized once every four weeks, five times. In the first immunization, ZEN-BSA was dissolved in sterilized PBS and emulsified with the same amount of FCA. In the enhanced follow-up immunization, ZEN-BSA was dissolved in sterilized PBS and fully emulsified with an equal amount of FIA, and this was administered four weeks after the first immunization. Later, 21 days after the last immunization, the tail was cut off for blood collection. The serum was separated to obtain ZEN pAb.

### 2.8. Identification of ZEN pAb

#### 2.8.1. Titers Measurement

The ZEN pAb titers were tested through indirect noncompetitive ELISA (inELISA) using the procedure described by Han et al. [34]. The coating antigen ZEN-OVA was diluted in CBS at 2 *μ*g/mL, and 100 mL/well was added to the 96-well microplate and incubated at 37.8°C for 2 h. After washing with PBST three times, unbound active sites were blocked with 250 *μ*L/well of blocking buffer at 37.8°C for 1 h or 4°C overnight. After subjecting the microplate to another wash, 50 mL/well of ZEN pAb with appropriate dilution was added and incubated for 15 min at 37.8°C. After another washing procedure, GaMIgG-HRP (50 mL/well) was added, followed by incubation for 30 min at 37.8°C. After washing six times, we added onto the microplate (60 *μ*L/well) freshly prepared TMB solution, followed by incubation at room temperature for 10 min. The reaction was stopped by adding 2 mol/L H_2_SO_4_ (100 mL/well), and then, measurements of absorbance were taken at 450 nm. Preimmunization serum and PBST was used as a negative control and blank control, respectively, which we included in all assays. The ZEN pAb titers were defined as the reciprocal of the dilution that resulted in an absorbance value, twice the blank value. Each ZEN pAb sample and negative sample were repeated for six times, and the average value was taken. Among the four groups of mice immunized with four immunogens, the mice with the highest titers in each group were selected for evaluating the immune effects of the four immunogens.

#### 2.8.2. Sensitivity Measurement

We determined the sensitivity at 50% inhibition concentration (IC50) of ZEN, whereas the IC50 value was determined via indirect competitive ELISA (icELISA) using the procedure described by Imtiaz and Yunus [35]. The icELISA method was the same as the inELISA except that, after blocking, a competition step was introduced by adding 50 mL/well of the analyte, followed by 50 mL/well of appropriate antibodies. The inhibition rate B/B0% of different concentrations of ZEN to ZEN pAb was assessed by icELISA, where B denoted the absorbance value of different ZEN concentrations, and B0 represented the absorbance value of ZEN 0 concentration. The inhibition curve was drawn using B/B0% as the ordinate and the logarithm of different ZEN concentrations as the abscissa. We then deduced the regression equation and calculated the IC50 for ZEN. Each ZEN pAb sample was repeated three times and the average value was taken. Among the four groups of mice immunized with four immunogens, the mice with the lowest IC50 value for ZEN in each group were selected to evaluate the sensitivity of the four ZEN pAbs.

#### 2.8.3. Specificity Measurement

We used the cross-reactions test (CR) as described by Ertekin et al. [36]. According to the CR, ZEN and its structural analogs, including *α*-ZAL, *β*-ZAL, *α*-ZOL, *β*-ZOL, and ZON, were selected as inhibitors. The IC50 values for ZEN and other inhibitors were determined by icELISA. The percentage of IC50 value for ZEN to IC50 value for each inhibitor was considered the cross-reaction percentage (CR%). The CR% was calculated using the formula: CR (%) = (IC50 (ZEN)/IC50 (ZEN analogs)) × 100%. One sample of ZEN pAb with the lowest IC50 value for ZEN was selected from the mice immunized with each immunogen for specificity determination. Each ZEN pAb sample was repeated for three times, and the average value was taken.

## 3. Results and Analysis

### 3.1. IR Identification

**Figure 6:**
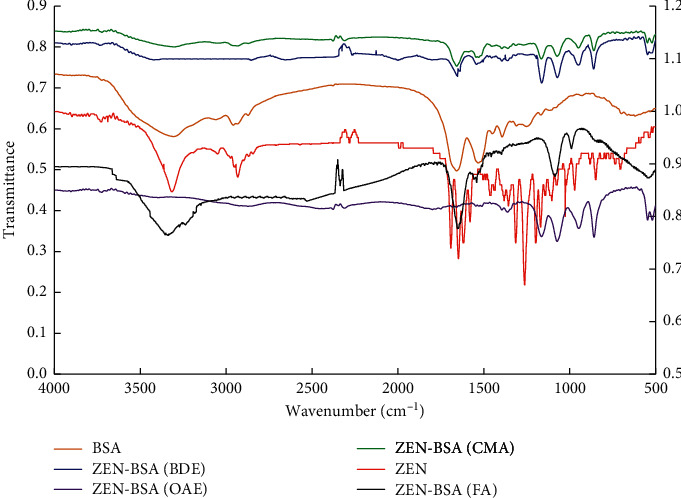
IR spectra of ZEN-BSA synthesized by four methods.

Comparing the IR of the artificial immunogen ZEN-BSA synthesized via the four methods with BSA, we revealed similar IR absorption in the regions 2800–3200 cm^−1^ and 1100–1700 cm^−1^, which were the characteristic peaks produced by the amine group and amide group in BSA (Figure 6). Notably, this proved that artificial immunogen ZEN-BSA synthesized by the four methods contains BSA. Moreover, both the IR of the artificial immunogen ZEN-BSA synthesized by the four strategies and ZEN had similar IR absorption in the regions 2200–2300 cm^−1^ and 1100–1400 cm^−1^. In contrast, BSA had no absorption in the same areas. The characteristic peaks produced by hydroxyl and esters in ZEN indicated that the artificial immunogen ZEN-BSA synthesized by the four methods contain ZEN. These findings revealed that the four methods successfully synthesized the artificial immunogen ZEN-BSA.

### 3.2. UV Identification

**Figure 7:**
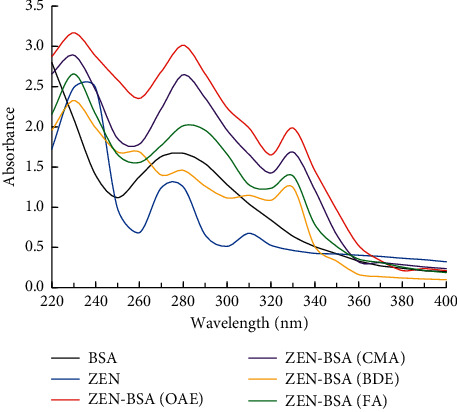
UV spectra of ZEN-BSA synthesized via the four methods.

In the range of UV 220–400 nm, BSA had characteristic absorption peaks for UV at 278 nm, and ZEN had characteristic absorption peaks for UV at 236 nm, 274 nm, and 316 nm, respectively (Figure 7). The artificial immunogen ZEN-BSA synthesized via the four methods (OAE, CMA, FA, and BDE) all showed the characteristic absorption peaks of BSA and ZEN, or the characteristic absorption peaks were shifted. Thus, the previously mentioned four methods could synthesize the artificial immunogen ZEN-BSA. According to the Lambert-Beer law, formula *A* = *εCL* (where *A* denotes the absorbance value read by the instrument; *ε* denotes the molar extinction coefficient, which is a constant value; *C* denotes the solute concentration in the solution; *L* represent the optical path, as determined by the instrument) was applied to calculate the molecular binding ratio of ZEN and BSA (see the results in Table 1).

### 3.3. SDS-PAGE Identification

**Figure 8:**
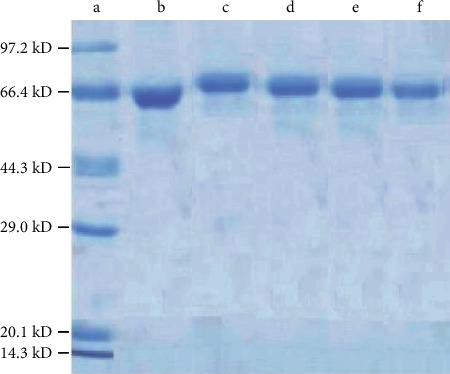
SDS-PAGE image of ZEN-BSA synthesized via the four methods. (a) Maker; (b) BSA; (c) ZEN-BSA (OAE); (d) ZEN-BSA (CMA); (e) ZEN-BSA (FA); (f) ZEN-BSA (BDE).

All the electrophoretic bands of the artificial immunogen ZEN-BSA synthesized via the four methods were lagging behind the BSA bands, indicating that the molecular weight of ZEN-BSA was greater than BSA. This proved the successful synthesis of ZEN-BSA (Figure 8). Detected by the gel imaging system, the molecular weight of BSA was 66446 Da, whereas the molecular weight of ZEN-BSA (OAE), ZEN-BSA (CMA), ZEN-BSA (FA), and ZEN-BSA (BDE) was 71803 Da, 70994 Da, 69482 Da, and 699023 Da, respectively. The molecular binding ratios of ZEN and BSA in ZEN-BSA (OAE), ZEN-BSA (CMA), ZEN-BSA (FA), and ZEN-BSA (BDE) were 17.17 : 1, 14.57 : 1, 9.73 : 1, and 8.26 : 1, respectively, which concurred with the UV identification results.

### 3.4. ZEN pAb Characteristic Analysis

#### 3.4.1. Titers Measurement

**Figure 9:**
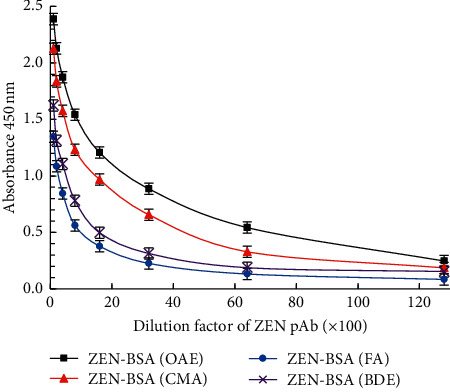
The inELISA titers measurement for ZEN pAb. Each point represents the mean of three replicates (*n* = 3).

After immunization, we selected one immunized mouse with the highest inELISA titers from each group for comparative analysis. The inELISA titers of immunized mice with ZEN-BSA (OAE), ZEN-BSA (CMA), ZEN-BSA (FA), and ZEN-BSA (BDE) were 1 : (6.4 × 10^3^), 1 : (3.2 × 10^3^), 1 : (1.6 × 10^3^) and 1 : (1.6 × 10^3^), all of which attained beyond 1 : (1 × 10^3^) (Figure 9). Following the previously mentioned results, the four artificial immunogens synthesized showed satisfactory immunogenicity. According to the inELISA titers, the immune effects of the four immunogens were evaluated in the order of ZEN-BSA (OAE), ZEN-BSA (CMA), ZEN-BSA (BDE), and ZEN-BSA (FA).

#### 3.4.2. Sensitivity Measurement

**Figure 10:**
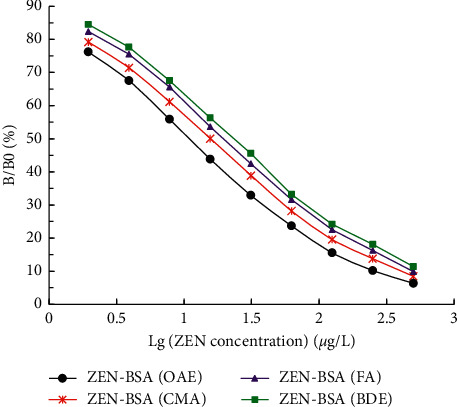
Sensitivity measurement of ZEN pAb to ZEN via icELISA. The values indicate the mean of three independent assays (*n* = 3).

The icELISA curves of the four immunized mice with the highest inELISA titers selected by each group showed a good linear relationship (Figure 10). The regression equation, *R*^2^ value, and IC50 value of the inhibition curve are shown in Table 2. The IC50 value for ZEN by icELISA of each immunogen ZEN-BSA (OAE), ZEN-BSA (CMA), ZEN-BSA (FA), and ZEN-BSA (BDE) were 11.67 *μ*g/L, 16.29 *μ*g/L, 20.92 *μ*g/L, and 24.36 *μ*g/L, respectively. Using IC50 value for ZEN, in evaluating the immune effect of the four artificial immunogens, the order was reported as follows: ZEN-BSA (OAE), ZEN-BSA (CMA), ZEN-BSA (FA), and ZEN-BSA (BDE).

#### 3.4.3. Specificity Identification

ZEN pAb produced by immunizing mice with ZEN-BSA (OAE), ZEN-BSA (CMA), ZEN-BSA (FA), and ZEN-BSA (BDE) could all recognize ZEN 100% (Table 3). The ZEN pAb from the mice immunized with ZEN-BSA (OAE) and ZEN-BSA (CMA) had similar characteristics; that is, it had class broad specificity for ZEN and its analogs. The IC50 value for ZEN by icELISA of the ZEN pAb from the mice immunized with ZEN-BSA (OAE) was 11.67 *μ*g/L, and the CR values of *α*-ZAL, *β*-ZAL, *α*-ZOL, *β*-ZOL, and ZON were 36.53%, 16.98%, 64.33%, 20.16%, and 10.66%, respectively. The IC50 value for ZEN by icELISA of the ZEN pAb from the mice immunized with ZEN-BSA (CMA) was 16.29 *μ*g/L, and the CR values of *α*-ZAL, *β*-ZAL, *α*-ZOL, *β*-ZOL, and ZON were 23.55%, 12.18%, 51.86%, 18.62%, and 9.64%, respectively. In terms of class broad-specificity, the ZEN pAb from mice immunized with ZEN-BSA (OAE) was more prominent. Furthermore, the ZEN pAb from the mice immunized with ZEN-BSA (FA) and ZEN-BSA (BDE) exhibited similar characteristics; that is, it had high specificity for ZEN but with poor class broad specificity for ZEN analogs. The IC50 value for ZEN by icELISA of the ZEN pAb from the mice immunized with ZEN-BSA (FA) was 20.92 *μ*g/L, and the CR values of *α*-ZAL, *β*-ZAL, *α*-ZOL, *β*-ZOL, and ZON were all less than 1%. The IC50 value for ZEN by icELISA of the ZEN pAb from the mice immunized with ZEN-BSA (BDE) was 24.36 *μ*g/L, except for the higher CR (36.57%) with ZON, and the CR values of *α*-ZAL, *β*-ZAL, *α*-ZOL, and *β*-ZOL were less than 3%. In terms of specificity, the ZEN pAb from mice immunized with ZEN-BSA (FA) was more prominent. These results demonstrated that the preparation of the class broad-specificity antibodies for ZEN and its analogs can be achieved by immunizing animals with the immunogen ZEN-BSA prepared by OAE method, while the preparation of highly specific antibodies for ZEN can be achieved by immunizing animals with the immunogen ZEN-BSA prepared by the FA method.

## 4. Discussion

### 4.1. Design of the ZEN Immunogen Synthesis Method

ZEN has a molecular weight of 318.36 Da and is a small molecule compound. It has only reactogenicity but no immunogenicity. It cannot directly induce the body to produce specific antibodies. It must be combined with the carrier protein to form an immunogen to induce the body to produce specific antibodies. Therefore, the design of ZEN immunogen synthesis method is very important [37]. According to the hapten-carrier effect theory, in the immunogen synthesis method, the main factors affecting the characteristics of the antibody produced by the immunogen include the complexity of the hapten molecular structure, selection of the position of the active site, length of the introduced spacer, molecular space structure, and molecular binding ratio of hapten to carrier protein [38]. The complexity of hapten molecular structure refers to whether the hapten molecular structure contained benzene ring and number, heterocycle and number, and branch structure and number. Generally, the hapten with complex structure easily induces the body to produce antibodies, while the hapten with simple structure difficultly induces the body to produce antibodies [39]. Szurdoki et al. reported that the success rate of preparing antibodies with a benzene ring in the hapten molecular structure was 33.3%, while the success rate without a benzene ring was only 9.1% [40]. Regarding the selection of active sites, on the one hand, different active sites will cause changes in the distribution of electrons in the hapten molecule, thereby changing its electrochemical properties, thereby changing its immune activity, and affecting the affinity of antibodies [33]. On the other hand, because different active sites will lead to different spatial structures of molecules, the exposed antigenic determinants are also different, which in turn affects the specificity of antibodies [41]. Therefore, we should try to use different sites to synthesize immunogens separately and then, through comparative analysis, screen out the best immunogen for target antibody preparation. The selection of the spacer arm includes the length and structure of the spacer arm. For the determination of the spacer arm length, Tuomola et al. and Jung et al. proposed that the optimal length of the spacer arm was three to six carbon atoms. Shortness of spacer arm was not conducive to full exposure of hapten, and too long spacer arm would cause the folding of alkyl chain due to hydrophobic effect, which would cause the hapten molecule to be covered by carrier protein, which was not conducive to be recognized by antigen-presenting cells [42, 43]. Regarding the choice of the spacer structure, Bruun et al. and Kolosova et al. considered that the spacer should not contain structures with high immunological activity such as aromatic rings, conjugated double bonds, or heterocycles, so as to reduce the production of antibodies. Overrecognition of the spacer arm reduced the ability to recognize the target molecule [44, 45]. Regarding the determination of the molecular binding ratio, Vikas et al. and Peterson et al. believed that when the molecular binding ratio was too large, the carrier protein was not conducive to the binding of the carrier protein and lymphocytes due to the excessive coverage of the hapten molecule, which weakened the carrier effect. If the molecular binding ratio was too small, the immunogen was not enough to cause the body to produce an immune response. The best molecular binding ratio of the hapten to the carrier protein should be 5–20 : 1 [46, 47].

In this study, based on the molecular structure characteristics of ZEN, and on the basis of a careful and comprehensive summary of previous studies, the C6′ position carbonyl group, C7′ position active hydrogen, and C2 position hydroxyl group were selected as active sites, and the spacer arm was introduced through different chemical reaction methods to realize the coupling of ZEN with carrier protein to synthesize immunogen.

### 4.2. Synthesis Methods of ZEN Immunogen and Characteristics of ZEN Antibody

ZEN belongs to the chemical compound of the resorcyclic acid lactones, which contains the main characteristic structure of 2,4-dihydroxybenzene ring and macrocyclic lactone ring. ZEN and its five analogs are structurally identical apart from minor differences at C6′ position carbonyl group or hydroxyl group and at C1′-C2′ position single bond or double bond in the macrocyclic lactone ring. Therefore, the special chemical properties of ZEN lay a good foundation for the preparation of class broad-specificity antibodies of ZEN and its five analogs but meanwhile also bring difficulties for the preparation of high-specificity antibodies of ZEN [30]. The specificity of antibody depends on the recognition of immunogen determinants by immune cells. The spatial conformation and characteristic structure exposure of hapten on carrier protein are closely related to the immune recognition of immune cells. Therefore, in hapten design, its main characteristic structure must be exposed as a part of the immunogen to the maximum extent in order to obtain the desired target antibody [29, 48]. Because of this, hapten molecular design, immunogen synthesis and ZEN high-specificity antibody preparation and ZEN class broad-specificity antibody preparation are the key to realize ZEN residual immunoassay and ZEN and its analogs total residual immunoassay. Several recent reports have used immunogens developed using the OAE method to obtain ZEN class broad-specificity antibodies and using FA method to obtain ZEN highly specific antibodies. A comparison between ZEN pAbs in this study and other ZEN pAbs or ZEN mAbs reported in the recent literature is shown in Table 4.

The OAE method is a classic and commonly used bioconjugation method. In recent years, some scholars have used this method to synthesize immunogens and have successfully prepared ZEN antibodies with class broad specificity [23, 27, 28, 49, 51]. Dong et al. [49], Zhang et al. [23], and Cha et al. [27] hold the same view on this. They believe that the OAE method is to introduce the carboxyl group onto the macrolide ring of ZEN at the C6′ position through the carbonyl oximation reaction, and then, the active ester is used to couple the carboxyl group with the amino group of carrier protein with a monoamide bond, so that the main characteristic structures of ZEN including 2,4-dihydroxybenzene ring, macrolide ring, carbonyl or hydroxyl group at C6′ position, and double bond at C1′-C2′ position can be fully exposed and recognized by immune cells, resulting in a strong immune response. The antibody produced can recognize ZEN and its five analogs. In addition, Liu et al. [28] and Dixon et al. [53] consider whether the double bond at the C6′ position and C1′-C2′ position of ZEN can be recognized by immune cells, and the degree of recognition determines the class broad specificity of ZEN antibody and has no direct relationship with the hydroxyl group at the C6′ position.

The FA method is also one of the classic biological coupling methods. Burkin et al. [52] and Gao et al. [29] have used this method to synthesize immunogens and have successfully prepared ZEN antibodies with high specificity [29, 52]. Burkin et al. [52] believe that the FA method is to introduce formyl substituents into the macrolide ring of ZEN at C7′ position through the formaldehyde condensation, so that the main characteristic structures of ZEN including the 2,4-dihydroxybenzene ring, macrolide ring, and carbonyl group at C6′ position are fully exposed, which are easier to be recognized by immune cells, and the antibody produced is highly specific to ZEN. However, the CRs for ZEN five analogs are small. As for why immune cells cannot recognize the single bond and double bond at C1′-C2′ position and the hydroxyl group at C6′ position, the reaction mechanism is still obscure.

In this study, the ZEN antibody with class broad specificity for ZEN and its five analogs was prepared by the OAE method, and the IC50 for ZEN was 11.67 *μ*g/L, and the CRs for *α*-ZAL, *β*-ZAL, *α*-ZOL, *β*-ZOL, and ZON were 36.53%, 16.98%, 64.33%, 20.16%, and 10.66%, respectively. However, the ZEN pAb had a lower broad specificity than that of ZEN mAb 6C2 reported by Dong et al. [49], ZEN mAb 3D4 reported by Zhang et al. [23], and ZEN mAb kk-ZEN reported by Cha et al. [27]. Compared with the OAE method, the CMA method had the defects of poor broad-specificity antibody, more side reactions, and lower product yield. The ZEN antibody with high-specificity for ZEN was prepared by the FA method, and the IC50 for ZEN was 20.92 *μ*g/L, and the CRs for *α*-ZAL, *β*-ZAL, *α*-ZOL, *β*-ZOL and ZON were all less than 1%. The antibody had high-specificity similar to that previously reported by Burkin et al. [52] and Gao et al. [29], but had better sensitivity. Compared with FA method, the antibody produced by the BDE method had too high CR with ZON, which could not meet the purpose of preparation of high-specificity antibody against ZEN. These results lay the material and technical foundation for the immunoassay of single residues for ZEN and total residues for ZEN and its analogs.

In addition, Li et al. believed that the specificity and broad specificity of antibodies not only depend on the synthesis method of immunogen but are also closely related to the animal immunization dose of the immunogen. Taking aflatoxin B1 as the research object, the effect of immunogen dose on antibody specificity and class broad specificity was discussed. The results showed that low-dose immunogen can improve the specificity of the antibody, and high-dose immunogen can increase the broad specificity of the antibody, and the research results provided reference for the development of new antibodies [54]. However, whether the results of this study are applicable to the preparation of antibodies to ZEN and other small molecule compounds remains to be further explored in the future.

## 5. Conclusion

In this study, according to the molecular structure and active sites of ZEN, four artificial immunogen synthesis methods were designed, and they were identified by IR, UV, SDS-PAGE, and comparative analysis of the characteristics of ZEN pAb produced by immunized animals. The results showed that the OAE method was the best method for preparing broad-specificity antibodies for ZEN and its homologs, and the FA method was the best method for preparing ZEN high-specificity antibodies, which laid a material and technical foundation for single immunoassay of ZEN residue and total amount immunoassay of ZEN and its analogs.

## Figures and Tables

**Table 1:** Molecular binding ratio of ZEN-BSA prepared via the four methods.

Synthesis methods	Initial molar ratio of ZEN to BSA	Molecular binding ratio of ZEN-BSA	Usage percentage of ZEN^∗^ (%)
OAE	50 ∶ 1	17.2 ∶ 1	34.4
CMA	50 ∶ 1	14.6 ∶ 1	29.2
FA	50 ∶ 1	9.7 ∶ 1	19.4
BDE	50 ∶ 1	8.3 ∶ 1	16.6

^∗^Since the molecular weight of BSA (66446) was much larger than that of ZEN (318.36), the utilization rate of BSA was set to 100% when calculating the utilization rate of BSA and ZEN.

**Table 2:** The regression equation, *R*^2^ and IC50 value for ZEN via icELISA.

Group	Regression equation	*R* ^2^	IC50 (*μ*g/L)
ZEN-BSA (OAE)	*y* = −30.589*x* + 82.637	0.9805	11.67
ZEN-BSA (CMA)	*y* = −31.071*x* + 87.658	0.9895	16.29
ZEN-BSA (FA)	*y* = −31.894*x* + 92.116	0.9918	20.92
ZEN-BSA (BDE)	*y* = −32.173*x* + 94.599	0.9922	24.36

**Table 3:** The cross-reaction of ZEN pAb with ZEN and its analogs.

Compound	ZEN pAb (OAE)		ZEN pAb (CMA)		ZEN pAb (FA)		ZEN pAb (BDE)	
	IC50 (*μ*g/L)^a,b^	CR (%)^a^	IC50 (*μ*g/L)^a,b^	CR (%)^a^	IC50 (*μ*g/L)^a,b^	CR (%)^a^	IC50 (*μ*g/L)^a,b^	CR (%)^a^
ZEN	11.67	100	16.29	100	20.92	100	24.36	100
*α*-ZAL	31.95	36.53	69.17	23.55	2682.05	0.78	1441.42	1.69
*β*-ZAL	68.73	16.98	133.74	12.18	2582.72	0.81	2511.34	0.97
*α*-ZOL	18.14	64.33	31.41	51.86	3670.18	0.57	990.24	2.46
*β*-ZOL	57.89	20.16	87.49	18.62	4358.33	0.48	1331.15	1.83
ZON	109.48	10.66	168.98	9.64	2273.91	0.92	66.61	36.57

^a^All of the data were calculated from triplicate assays. ^b^The compound standard solution was prepared in 70% methanol-PBS (7 : 3, v/v).

**Table 4:** Comparison of IC50 values and CR values of ZEN pAbs by the OAE method and FA method in this study and other previously reported ZEN pAbs and ZEN mAbs.

References	ZEN antibody	Coupling method	Immunoassay format	IC50 of ZEN (*μ*g/L)	CR (%)^a^				
					*α*-ZAL	*β*-ZAL	*α*-ZOL	*β*-ZOL	ZON
This study (2021)	ZEN pAb	OAE	icELISA^b^	11.67	36.53	16.98	64.33	20.16	10.66
Dong et al. [49]	ZEN mAb 6C2	OAE	icELISA	114.0	99.2	55.5	89.5	39.3	—
Zhang et al. [23]	ZEN mAb 3D4	OAE	FPIA^c^	0.041	107.9	57.2	103.5	73.9	66
Liu et al. [50]	ZEN mAb 7B2	OAE	BA-ELISA^d^	0.18	46.7	39.2	60.5	24.7	59.5
Cha et al. [27]	ZEN mAb kk-ZEN	OAE	icELISA	131.3	108.1	119.3	114.1	130.3	—
			dcELISA^e^	150.6	123.8	99.3	149.5	124.8	—
Thongrussamee et al. [51]	ZEN mAb 2C5	OAE	dcELISA	1.79	121.5	65.3	21.5	18.9	—
This study (2021)	ZEN pAb	FA	icELISA	20.92	0.78	0.81	0.57	0.48	0.92
Burkin et al. [52]	ZEN pAb	FA	icELISA	31.7	0.12	—	0.15	0.02	—
Gao et al. [29]	ZEN pAb	FA	icELISA	233.35	2.25	5.65	3.14	1.96	6.79
	ZEN mAb^#^	FA	icELISA	55.72	0.63	0.92	0.65	0.94	1.48

^a^All of the data were calculated using the CR of ZEN as 100%. ^b^icELISA: indirect competitive enzyme-linked immunosorbent assay; ^c^FPIA: fluorescence polarization immunoassay; ^d^BA-ELISA: biotin-streptavidin amplified enzyme-linked immunosorbent assay; ^e^dcELISA: direct competitive enzyme-linked immunosorbent assay; —: no data. ^#^No name.

## Data Availability

No data were used to support this study.
